# Bariatric surgery volume by hospital and long-term survival: population-based NordOSCo data

**DOI:** 10.1093/bjs/znac381

**Published:** 2022-11-16

**Authors:** Sheraz R Markar, Giola Santoni, Dag Holmberg, Joonas H Kauppila, Jesper Lagergren

**Affiliations:** Department of Molecular Medicine and Surgery, Karolinska Institutet, Karolinska University Hospital, Stockholm, Sweden; Department of Surgery and Cancer, Imperial College London, London, UK; Nuffield Department of Surgery, Oxford University, Oxford, UK; Department of Molecular Medicine and Surgery, Karolinska Institutet, Karolinska University Hospital, Stockholm, Sweden; Department of Molecular Medicine and Surgery, Karolinska Institutet, Karolinska University Hospital, Stockholm, Sweden; Department of Molecular Medicine and Surgery, Karolinska Institutet, Karolinska University Hospital, Stockholm, Sweden; Surgery Research Unit, University of Oulu and Oulu University Hospital, Oulu, Finland; Department of Molecular Medicine and Surgery, Karolinska Institutet, Karolinska University Hospital, Stockholm, Sweden; School of Cancer and Pharmaceutical Sciences, King’s College London, London, UK

## Abstract

**Background:**

It is unclear whether annual hospital volume of bariatric surgery influences the long-term survival of individuals who undergo surgery for severe obesity. The hypothesis that higher annual hospital volume of bariatric surgery is associated with better long-term survival was evaluated.

**Methods:**

This retrospective population-based study included patients who underwent bariatric surgery in Sweden and Finland between 1989 and 2020. Annual hospital volume was analysed for risk of all-cause mortality. Multivariable Cox regression provided HRs with 95 per cent confidence intervals adjusted for age, sex, co-morbidity, country, and type of bariatric procedure.

**Results:**

Weight loss surgery was performed in 77 870 patients with a 0.5 per cent risk of postoperative death (mortality rate (MR) per 100 000 people 592.7, 95 per cent c.i. 575.0 to 610.9). Higher annual hospital volume of bariatric surgery was associated with a lower risk of all-cause mortality. The adjusted HRs were slightly more reduced for each quartile of annual hospital volume compared with the lowest quartile (MR per 100 000 people for lowest quartile 815.1, 95 per cent c.i. 781.7 to 849.9; for quartile II: HR 0.88, 95 per cent c.i. 0.81 to 0.96 (MR per 100 000 people 545.0, 512.0 to 580.1); for quartile III: HR 0.87, 0.78 to 0.97 (MR per 100 000 people 428.8, 395.5 to 465.0); for quartile IV: HR 0.82, 0.73 to 0.93 (MR per 100 000 people 356.0, 324.1 to 391.1)). In analyses restricted to laparoscopic surgery, volume and mortality were related only in the crude model (HR 0.86, 0.75 to 0.98), but not in the multivariable model (HR 0.97, 0.84 to 1.13) that compared highest and lowest quartiles.

**Conclusion:**

If there was a survival benefit associated with hospital volume, it may have been due to a faster uptake of laparoscopic surgery in the busier hospitals.

## Teaser

Currently there is a paucity of evidence concerning the influence of hospital volume on prognosis after bariatric surgery. This population-based cohort study, which included over 75 000 patients from Sweden and Finland who underwent bariatric surgery, suggests that higher annual hospital volume improves long-term survival.

## Introduction

Bariatric surgery in severely obese adults aims to induce weight loss, reduce co-morbidities, and improve quality of life, but many patients experience weight regain^1–5^. A worrying trend in bariatric operations underlines the expanding problem: even if they do reduce mortality^6–9^ and medication use (for example treatment of diabetes)^10^, the cost is considerable. Does a mere 25 per cent loss of excess weight and 38 per cent reduction in medication use balance the initial high cost of preoperative care, operation, and considerable rehabilitation?^11^ Published outcomes, with a reintervention rate of 2.2 per cent and 90-day mortality rate of 0.2 per cent, are from centres of excellence^10^, but do these reflect real-world data outside such centres?

The prognostic role of higher hospital volume has been established for some gastrointestinal operations (such as oesophagectomy and pancreatectomy)^12–14^, for which the main differences relate to teamwork, decision-making, and patient selection^15,16^. A meta-analysis^17^ suggested that higher hospital volume of bariatric surgery was associated with reduced short-term mortality, yet the resolution for detecting differences belies the reportedly low rate of death. This study addressed this conflict using population-based data and long-term survival.

## Methods

### Design

A population-based study was designed (data from 1989–2020) to examine how annual hospital volume of bariatric surgery influences all-cause mortality using the Nordic Obesity Surgery Cohort (NordOSCo) data set^18^. Reporting of patient admission, diagnosis codes, and surgical procedures to the national health data registries is mandatory^19^. Each resident is assigned a unique personal identity code at birth (or immigration) which is used for governmental and research purposes^20,21^. The personal identity code system allows accurate linkage of information between registries for each individual^19^. The study followed a detailed predefined study protocol, and was approved by the relevant ethical committees, data inspectorates, and governmental agencies managing the registries in Sweden and Finland^18^.

### Cohort

The Swedish and Finnish patient registries hold nationwide information on in-hospital care and outpatient specialist care, including diagnoses and surgical procedures^21,22^. In the present study, records of all patients from Sweden or Finland with an obesity diagnosis who underwent bariatric surgery during the study interval (1 January 1989 to 31 December 2020 in Sweden and 31 December 2018 in Finland) were examined. A validation study specifically of bariatric surgery in the Swedish Patient Registry had a positive predictive value of 97 per cent compared with the medical records of 938 patients^23^. Follow-up started on the first date of primary bariatric surgery and lasted until death or the end of the study interval, whichever occurred first^4^. The two countries shared indications and clinical guidelines for bariatric surgery.

### Exposure: annual hospital volume

The primary exposure was annual hospital volume of bariatric surgery categorized into four groups of equal size (quartiles). Quartile I included hospitals conducting 55.5 or fewer, quartile II 55.75–111.25, quartile III 111.5–221.5, and quartile IV over 221.5 bariatric procedures per year. Secondary exposures were annual hospital volume categorized into 10 groups of about equal size (deciles) as well as considered as a continuous variable. To account for temporary fluctuations in annual hospital volume between calendar years, and to incorporate the experience achieved during the last few years before each operation, hospital volume was calculated using a 4-year moving average number of operations at each hospital. Thus, each patient was allocated the moving average of hospital volume for the year of their operation plus 3 years earlier. For example, for an operation conducted in 2005, the exposure was the average (mean) hospital volume at that hospital during the 4 years 2002, 2003, 2004, and 2005. During the first years of the study, when data from the previous 4 years did not exist, the available years were included; that is, for 1989, the hospital volume variable was calculated in that year, for 1990 it was calculated as an average across 1989 and 1990, and so on.

### Outcome: all-cause mortality

The outcome was all-cause mortality, defined as death from any cause. These data came from the national registries for mortality in Sweden and Finland, which are 100 per cent complete for date of death.

### Co-variables

Five co-variates were considered: patient age (continuous), designated sex (male or female), country (Sweden or Finland), co-morbidity (Charlson Co-morbidity Index score 0, 1, or 2 and above)^24^, and type of bariatric surgical procedure (gastric bypass, sleeve gastrectomy, gastric banding, vertical banded gastroplasty, or other). All co-variates were indexed on the date of study entry (date of bariatric surgery).

### Statistical analysis

Absolute mortality was presented as annual risk^25^ and in the form of Kaplan–Meier curves. Cox regression was used to calculate crude and adjusted HRs with 95 per cent confidence intervals^26^. The multivariable model adjusted for the five co-variates with the categorizations shown above. The proportionality of the hazard was tested using Schoenfeld residuals. Analyses stratified by follow-up time are also reported because the proportionality was not met in the main model. Subgroup analyses included analyses restricted to laparoscopic bariatric surgery, and those stratified by each of the five co-variates: age (2 groups divided by the mean value), sex (male and female), country (Sweden and Finland), co-morbidity (Charlson Co-morbidity index 0, 1, and 2 or higher), and type of bariatric procedure (gastric bypass and all other procedures). There were no missing data, so complete-case analysis was used.

## Results

### Patients

**Fig. 1:**
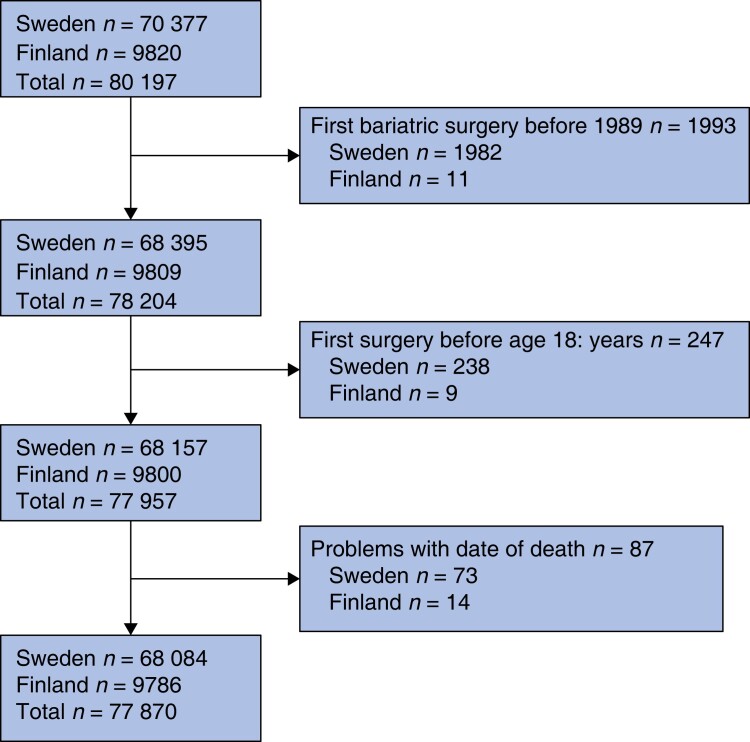
Study flow chart

*Figure 1* shows the selection of patients for the study. After exclusions, 77 870 patients remained for final analysis, including 68 084 (87.4 per cent) from Sweden and 9786 (12.6 per cent) from Finland. The age, assigned sex, and co-morbidity distributions were similar in patients in the four annual hospital volume quartiles (*Table 1*). Gastric bypass, sleeve gastrectomy, and a laparoscopic approach were performed more in high- than in low-volume centres.

**Table 1:** Characteristics of patients in Swedish and Finnish bariatric surgery cohorts (77 870 patients)

	Quartile of annual hospital volume			
	I	II	III	IV
No. of patients	19 471 (25.0)	19 548 (25.1)	19 514 (25.1)	19 337 (24.8)
Hospital volume, median (i.q.r.)	29.0 (15.3–42.8)	80.8 (68.5–96.5)	161.0 (133.0–188.8)	312.8 (249.8–480.5)
Age (years), median (i.q.r.)	42 (34–50)	42 (33–50)	42 (33–50)	41 (33–50)
Female	14 507 (74.5)	14 726 (75.3)	14 513 (74.4)	14 734 (76.2)
**Charlson Co-morbidity Index score**				
ȃ0	14 110 (72.5)	13 436 (68.7)	13 577 (69.6)	14 189 (73.4)
ȃ1	4219 (21.7)	4769 (24.4)	4657 (23.9)	4136 (21.4)
ȃ≥ 2	1142 (5.9)	1343 (6.9)	1280 (6.6)	1012 (5.2)
**Type of surgery**				
ȃGastric bypass	11 196 (57.5)	15 283 (78.2)	16 049 (82.2)	15 435 (79.8)
ȃSleeve gastrectomy	1948 (10.0)	2363 (12.1)	3047 (15.6)	3695 (19.1)
ȃVertical banded gastroplasty	2636 (13.5)	721 (3.7)	51 (0.3)	0 (0.0)
ȃGastric banding	2966 (15.2)	912 (4.7)	15 (0.1)	4 (<0.1)
ȃOther restrictive procedures	313 (1.6)	143 (0.7)	11 (0.1)	18 (0.1)
ȃBlocking procedures	412 (2.1)	126 (0.6)	341 (1.8)	185 (1.0)
Laparoscopic approach	10 840 (55.7)	16 430 (84.1)	18 681 (95.7)	19 009 (98.3)
**Calendar year**				
ȃMedian (i.q.r.)	2007 (1989–2019)	2012 (2009–2012)	2013 (2011–2016)	2014 (2012–2016)
ȃRange	1989–2019	1992–2019	1999–2019	2008–2019
**Country**				
ȃSweden	15 117 (77.6)	16 210 (82.9)	17 655 (90.5)	19 103 (98.8)
ȃFinland	4354 (22.4)	3338 (17.1)	1860 (9.5)	234 (1.2)
Deaths	2196 (11.3)	985 (5.0)	586 (3.0)	435 (2.3)
Person-time (years)	269 408	180 739	136 652	122 181
**Follow-up time**				
ȃMedian (i.q.r.)	12.2 (7.7–20.5)	8.1 (5.1–11.6)	7.1 (4.3–9.3)	6.6 (4.0–8.7)
ȃMean(s.d.)	13.8 (8.6)	9.3 (6.0)	7.0 (3.4)	6.3 (2.9)

Values are *n* (%) unless otherwise indicated. Quartile I, 55.5 or fewer; quartile II, 55.75–111.25; quartile III, 111.5–221.5; and quartile IV, more than 221.5 bariatric procedures annually.

### Hospital volume and all-cause mortality

**Fig. 2:**
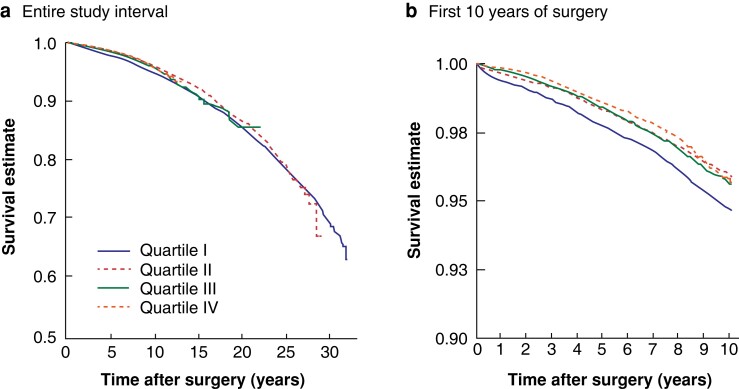
Kaplan–Meier curves for overall survival by annual hospital volume of bariatric surgery **a** Entire study interval and **b** first 10 years after surgery. Quartile I, 55.5 or fewer; quartile II, 55.75–111.25; quartile III, 111.5–221.5; and quartile IV, more than 221.5 bariatric procedures annually. Circle is point estimate and error bars are confidence interval.

**Fig. 3:**
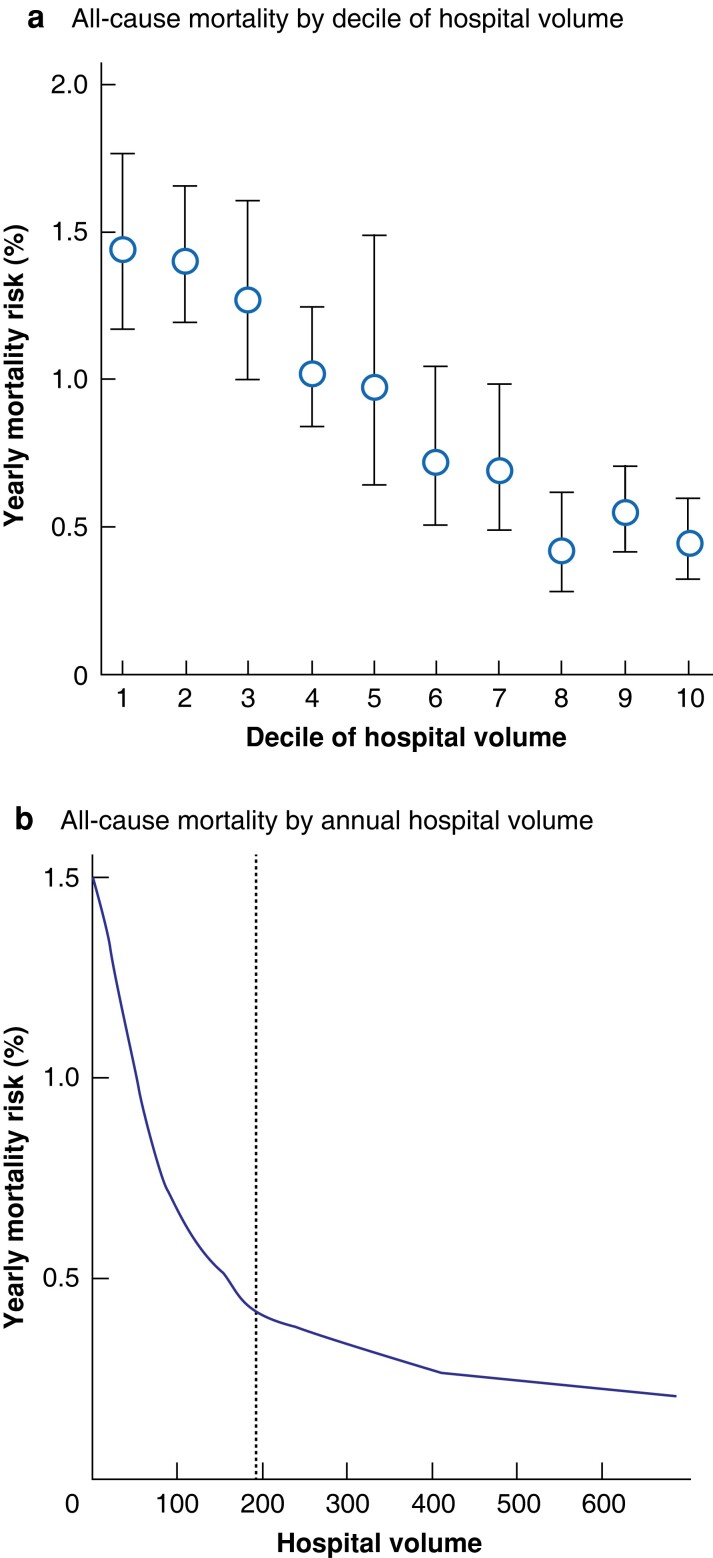
All-cause mortality in relation to annual hospital volume of bariatric surgery **a** All-cause mortality by decile of annual hospital volume of bariatric surgery. Decile 1, fewer than 24; decile 2, 24–44; decile 3, 45–65; decile 4, 66–86; decile 5, 86.25–111.25; decile 6: 111.5–151; decile 7, 151.5–192.25; decile 8, 192.75–245; decile 9, 246–367.75; and decile 10, 369.25–677.5 bariatric procedures annually. **b** All-cause mortality by annual hospital volume of bariatric surgery. The dotted line indicates the start of the eighth decile.

The annual mortality risk was inversely related to hospital volume. The mortality ranged from X to Y per cent for hospitals, with a median of Z per cent. There was a stepwise drop in mortality rate with increasing volume by quartile (quartile I, 1.43 (95 per cent c.i. 1.22 to 1.69) per cent; quartile II, 1.37 (0.92 to 2.05) per cent; quartile III, 0.71 (0.49 to 1.03) per cent; quartile IV, 0.53 (0.41 to 0.68) per cent) (*Fig. 2*). This reduction in annual mortality risk was most marked within the first 10 years of surgery (*Fig. 2*). When hospital volume was analysed in deciles, a stepwise reduction in percentage risk of mortality was observed, but a plateau was observed at deciles 8–10 (*Fig. 3*).

Increasing hospital volume was associated with reduced HRs for all-cause mortality. The adjusted HRs were slightly more reduced for each higher quartile of hospital volume compared with the lowest quartile (*Table 2*).

**Table 2:** Main analyses: HRs for all-cause mortality by annual hospital volume of bariatric surgery

Hospital volume	Mortality rate per 100 000 people	HR	
		Unadjusted model	Adjusted model*
Continuous		0.99 (0.99, 0.99)	0.99 (0.99, 1.00)
**Quartile**			
ȃI	815.1 (781.7, 849.9)	1.00 (reference)	1.00 (reference)
ȃII	545.0 (512.0, 580.1)	0.88 (0.82, 0.96)	0.88 (0.81, 0.96)
ȃIII	428.8 (395.5, 465.0)	0.89 (0.80, 0.98)	0.87 (0.78, 0.97)
ȃIV	356.0 (324.1, 391.1)	0.78 (0.70, 0.87)	0.82 (0.73, 0.93)

Values in parentheses are 95% confidence intervals. *Adjusted for age, sex, country, co-morbidity, and type of bariatric surgery.

The results of all subgroup analyses are shown in *Table 3*. In analyses restricted to laparoscopic surgery, increasing hospital volume was associated with reduced mortality only in the crude model (HR 0.86, 95 per cent c.i. 0.75 to 0.98), but not in the multivariable model (HR 0.97, 0.84 to 1.13), for the highest compared with the lowest quartile. Results for patients who had gastric bypass were similar to those of the entire group (adjusted HR 0.81, 0.71 to 0.93, for highest *versus* lowest quartile) and the corresponding HR for other types of bariatric surgery was decreased (adjusted HR 0.69, 0.52 to 0.92). All follow-up times after bariatric surgery showed decreased point estimates in the highest *versus* lowest quartile of hospital volume, but all HRs were consistently and statistically significantly decreased only for the group with follow-up of 7 years or less (adjusted HR 0.74, 0.64 to 0.86, for highest *versus* lowest quartile).

**Table 3:** Stratified analyses: HRs for all-cause mortality by annual hospital volume of bariatric surgery

	HR	
	Unadjusted model	Adjusted model*
**Laparoscopic bariatric surgery**		
ȃHospital volume	1.00 (0.99, 1.00)	1.00 (0.99, 1.00)
ȃȃQuartile I	1.00 (reference)	1.00 (reference)
ȃȃQuartile II	0.85 (0.75, 0.96)	0.90 (0.79, 1.03)
ȃȃQuartile III	0.94 (0.83, 1.07)	1.00 (0.88, 1.15)
ȃȃQuartile IV	0.86 (0.75, 0.98)	0.97 (0.84, 1.13)
**Type of bariatric surgery**		
ȃGastric bypass		
ȃȃQuartile I	1.00 (reference)	1.00 (reference)
ȃȃQuartile II	0.80 (0.71, 0.89)	0.84 (0.75, 0.93)
ȃȃQuartile III	0.85 (0.75, 0.95)	0.90 (0.80, 1.02)
ȃȃQuartile IV	0.73 (0.65, 0.83)	0.81 (0.71, 0.93)
ȃOther type		
ȃȃQuartile I	1.00 (Reference)	1.00 (Reference)
ȃȃQuartile II	0.99 (0.89, 1.11)	0.92 (0.82, 1.03)
ȃȃQuartile III	0.92 (0.73, 1.17)	0.63 (0.50, 0.81)
ȃȃQuartile IV	0.89 (0.67, 1.18)	0.69 (0.52, 0.92)
**Time after bariatric surgery**		
ȃ≤ 7 years		
ȃȃHospital volume	0.99 (0.99, 0.99)	0.99 (0.99, 1.00)
ȃȃȃ Quartile I	1.00 (reference)	1.00 (reference)
ȃȃȃ Quartile II	0.80 (0.70, 0.91)	0.82 (0.72, 0.94)
ȃȃȃ Quartile III	0.79 (0.69, 0.90)	0.81 (0.71, 0.93)
ȃȃȃ Quartile IV	0.66 (0.58, 0.76)	0.74 (0.64, 0.86)
ȃ7–11 years		
ȃȃHospital volume	1.00 (0.99, 1.00)	1.00 (0.99, 1.00)
ȃȃȃ Quartile I	1.00 (reference)	1.00 (reference)
ȃȃȃ Quartile II	0.77 (0.65, 0.91)	0.74 (0.63, 0.90)
ȃȃȃ Quartile III	0.91 (0.75, 1.10)	0.85 (0.70, 1.04)
ȃȃȃ Quartile IV	0.97 (0.79, 1.20)	0.91 (0.72, 1.14)
ȃ> 11 years		
ȃȃHospital volume	1.00 (0.98, 1.01)	0.99 (0.97, 1.01)
ȃȃȃ Quartile I	1.00 (reference)	1.00 (reference)
ȃȃȃ Quartile II	1.02 (0.91, 1.15)	1.01 (0.89, 1.13)
ȃȃȃ Quartile III	1.19 (0.87, 1.63)	1.01 (0.73, 1.41)
ȃȃȃ Quartile IV	1.17 (0.48, 2.84)	0.80 (0.32, 1.96)

Values in parentheses are 95% confidence intervals. *Adjusted for age, sex, country, co-morbidity, and type of bariatric surgery.

## Discussion

The population-based design (long follow-up and large sample size) may provide results that are valid and statistically robust, but limitations include the lack of data on preoperative BMI and postoperative weight change. Although adjustment was made for Charlson Co-morbidity Index score in the analyses, data on changes in medical co-morbidities after surgery were not available. Changes in bariatric surgical practice during the time interval included abandonment of some surgical techniques (for example vertical banded gastroplasty), introduction of others (for example sleeve gastrectomy), and more minimally invasive approaches. Thus, this study may to a some degree reflect practice in low-volume hospitals where updated changes may be restricted due to the existent challenges. However, changes in surgical techniques and postoperative BMI changes or co-morbidities during the study period are more likely mediators than confounders of the association between hospital volume and all-cause mortality. Another source of error is chance because the low all-cause mortality (mainly young adults) reduced the statistical power, which was evident in some of the subgroup analyses.

This study focused on hospital volume, whereas surgeon volume and experience were not captured. Surgeon volume is often correlated with hospital volume and may be a mediator or a confounder, which helps to explain associations. Centralization of certain complex surgical interventions to high-volume centres improves morbidity and mortality for a variety of possible reasons^12–16^. The next step in these volume–outcome investigations may be to identify the factors that mediated improved outcomes seen in high-volume centres. Bariatric surgical high-volume centres typically have a multidisciplinary team for psychological support, nutritional advice, and the assessment of operative risk, co-morbidities, and surgical performance^27^.

The subgroup analyses of laparoscopic surgery showed no association between hospital volume and all-cause mortality. These results may be misleading. It may be the strong correlation between hospital volume and laparoscopic surgery that drives the overall volume-mortality relationship observed. All high-volume centres used a laparoscopic technique, leaving no range of exposure in this subanalysis. This was not adjusted for in the multivariable model because of the strong correlation between laparoscopic surgery and calendar year with hospital volume.

## Data Availability

The NordOSCo data set is available from public repository upon request.
